# Injury prevalence and associated factors among Japanese lacrosse collegiate athletes

**DOI:** 10.3389/fspor.2024.1360639

**Published:** 2024-03-05

**Authors:** Takeshi Kimura, Aleksandra Katarzyna Mącznik, Akira Kinoda, Yuichi Yamada, Yuki Muramoto, Yoshinori Katsumata, Kazuki Sato

**Affiliations:** Institute for Integrated Sports Medicine, School of Medicine, Keio University, Tokyo, Japan

**Keywords:** lacrosse, sports injuries, sports injury epidemiology, college athletes, athletic injuries, cross-sectional survey, surveillance, injury prevalence

## Abstract

**Context:**

Sports injuries have a substantial impact on athletes' performance and health. To reduce the risk of an injury occurring, the prevalence, localization, and severity need to be established.

**Objective:**

To examine the prevalence of sports injuries in collegiate lacrosse athletes.

**Design:**

Descriptive epidemiological study using online survey design.

**Setting:**

Japanese universities associated with UNIVAS.

**Participants:**

A total of 1,689 Japanese collegiate lacrosse athletes, 978 females and 701 males.

**Main outcome measures:**

Athletes were surveyed on the injuries within the previous year, their severity, localization, and onset characteristics. The support of an athletic trainer and its association with the odds of sustaining an injury was assessed. Factors related to injuries were explored.

**Results:**

One-year prevalence of injuries was 42%. Male sex, higher year at the university, and support from an athletic trainer were identified as factors related to higher odds of sustaining an injury and practicing at least 5 days per week was associated with lower odds of sustaining an injury.

**Conclusions:**

Male sex athletes, and athletes at the higher year at university are especially at risk of sustaining a lacrosse injury. The aspects of training (e.g., frequency, volume) should be investigated across the athlete development process to address these findings. Further investigation is needed to determine the extent to which the support of athletic trainers affects both the frequency and severity of injuries in lacrosse athletes.

**Key points:**

- Japanese collegiate lacrosse athletes reported 42% 1-year-prevalence of sports injuries, with 82.3% of the injuries causing time lost from training and competition.
- Training parameters (e.g., frequency, volume) should be assessed in the light of long-term athlete development.
- Support from athletic trainers should be optimized to assist with reducing the number and severity of injuries both in short- and long-term.

## Introduction

Lacrosse is a contact sport involving considerable amount of running, changing direction, and throwing. The use of sticks to propel the ball and the dynamic and unpredictable nature of this sport produces a unique context for injuries' occurrence. Injuries in lacrosse are frequent and require time off training (1). A range of protective measures, including compulsory and optional use of protective equipment, has been introduced to reduce the risks but with limited success especially for concussions (1).

Lacrosse is contested slightly differently in males and females, including varying rules on protective equipment which may influence injury patterns (2). The most frequent lacrosse-related injuries in men include lower limb and upper limb, and lower limb and head/neck in women (1, 3). It has been previously reported that lacrosse female athletes have a higher risk of ACL (anterior cruciate ligament) injuries due to their upright posture and extended knees while they play (4). Females are also at higher risk of sustaining a head, face, and eye injury than males in lacrosse (5). Moreover, the frequency of injuries has been reported to increase from youth to junior to senior lacrosse levels indicating the age as a contributing factor (6). All these data come from lacrosse athletes in the US (United States of America), therefore perspectives from other countries are desperately needed to establish which injury patterns arise from the sport itself and which ones have a cultural context. This will allow future injury-prevention strategies to target cultural and sports-specific contributions explicitly, likely increasing their effectiveness.

The purpose of the current study was to establish the 1-year prevalence of, and the factors related to lacrosse-related injuries in Japanese collegiate athletes.

## Methods

### Research design

In this observational study with a cross-sectional design, data was collected using a web-based survey from June to October 2022. The study received approval from Keio University's Ethics Committee (approval number: 20211158). The study was conducted in compliance with the Declaration of Helsinki's principles and reported as per the STROBE-SIIS consensus statement and CROSS consensus statement (7–9).

### Participant recruitment

Potential participants were invited from the lacrosse teams associated with UNIVAS (Japan Association for University Athletics and Sport) and located across Japan. Athletes were then asked to read the study's information sheet and consented to participating in the survey. The sample size was calculated at 372 participants using expected proportion in the population from Japanese collegiate handball players (10) and the formula devised by Charan and Biswas (11).

### Survey

The survey was hosted on a website specifically created for this study (https://enquete.cc/q/BC2XC8A8). The anonymous survey asked about athlete characteristics, lacrosse experience, and injuries sustained while playing lacrosse in the previous year (retrospective design). Follow-up questions about the three most serious injuries included details on the injury location, severity, type, time of onset, mechanism, time lost from sports participation, and diagnosis. Questions were adapted from the Japanese Society of Clinical Sports Medicine and Japanese Society for Athletic Training consensus document and modified to suit collegiate athletes (12).

Injury severity was determined based on the amount of time lost from training/competition and classified using recent guidelines (8, 13) and consisted of: minimal—0 days missed; mild—1 day–1 week lost; moderate—1 week–1 month lost; severe—1 month–6 months lost, very severe—more than 6 months lost. The injury type was classified as new, recurrent (the injury disappeared before but occurred again), or exacerbated (symptoms worsened), while the injury mechanism was classified as direct (direct application of the force), indirect (indirect application of the force), or non-contact (12).

### Statistical analysis

The SPSS software [IBM Corp. Released 2021. IBM SPSS Statistics for Macintosh, Version 28.0. Armonk, NY: IBM Corp] was used for statistical analysis. The study gathered both qualitative and quantitative information. Continuous data was summarized using mean and standard deviation while discrete data was summarized by calculating counts and percentages. A *p*-value of less than 0.05 was considered to be statistically significant.

To calculate the one-year period prevalence, the number of injured athletes was divided by the total number of athletes and multiplied by 100%. We used the chi-square test (Pearson's chi-square, Fisher's exact test, or Fisher's exact test with Monte Carlo estimates, as appropriate) to compare the differences in injury characteristics such as location, severity, type, onset, mechanism, and time lost between females and males.

To assess the factors associated with injury occurrence, we utilized regression models that included participant demographics and sports participation. Multivariable logistic regression was used to estimate odds ratios (ORs) with 95% confidence intervals for each outcome variable.

## Results

### Participants

Characteristics of lacrosse athletes are presented in Table 1. Out of 1,689 athletes who responded to the survey, 42% reported at least one injury within the previous year, and 7% reported three or more injuries.

**Table 1:** Characteristics of Japanese collegiate lacrosse athletes.

Characteristic	All	Males	Females	Sex unspecified
	*n* = 1,689	*n* = 701	*n* = 978	*n* = 10
	100%	41.5%	57.9%	0.6%
Age, years; mean ± SD	19.9 ± 1.4	20.0 ± 1.4	19.8 ± 1.4	20.0 ± 1.4
Year at university; *n* (%)				
Year 1	552 (32.7)	228 (32.5)	322 (32.9)	2 (20.0)
Year 2	431 (25.5)	179 (25.5)	248 (25.4)	4 (40.0)
Year 3	264 (15.6)	113 (16.1)	149 (15.2)	2 (20.0)
Year 4	442 (26.2)	181 (25.8)	259 (26.5)	2 (20.0)
Lacrosse experience, years; mean ± SD	2.2 ± 1.4	2.1 ± 1.4	2.3 ± 1.4	2.1 ± 1.2
Supported by an athletic trainer	895 (53.0)	414 (59.1)	479 (49.0)	2 (20)
Height, cm; mean ± SD	164.6 ± 8.5	172.4 ± 5.5	159.0 ± 5.2	159.4 ± 5.2
Weight, kg; mean ± SD	59.0 ± 9.8	67.5 ± 7.6	53.0 ± 5.9	53.6 ± 7.0
BMI; mean ± SD	21.7 ± 2.2	22.7 ± 2.1	20.9 ± 1.9	21.1 ± 2.7
Practice days per week; mean ± SD	4.5 ± 0.8	4.8 ± 0.8	4.4 ± 0.8	3.9 ± 0.3
Matches/competitions per season; *n* (%)				
1–5 matches	1,076 (63.7)	331 (47.2)	736 (75.3)	9 (90.0)
6–10 matches	398 (23.6)	188 (26.8)	209 (21.4)	1 (10.0)
11–15 matches	106 (6.3)	86 (12.3)	20 (2.0)	0 (0.0)
16–20 matches	55 (3.3)	44 (6.3)	11 (1.1)	0 (0.0)
21–25 matches	27 (1.6)	25 (3.6)	2 (0.2)	0 (0.0)
26–30 matches	9 (0.5)	9 (1.3)	0 (0.0)	0 (0.0)
>30 matches	18 (1.1)	18 (2.6)	0 (0.0)	0 (0.0)
Reported injuries within a year; *n* (%)				
None	983 (58.2)	348 (49.6)	631 (64.5)	4 (40.0)
One	396 (23.5)	206 (29.4)	189 (19.3)	1 (10.0)
Two	188 (11.1)	85 (12.1)	100 (10.2)	3 (30.0)
Three or more	122 (7.2)	62 (8.9)	58 (6.0)	2 (20.0)

### Injuries

Lacrosse athletes (*n* = 1,689) sustained 1,138 injuries, with 82.3% of the injuries causing time lost from training and/or competition, 1 in 4 injuries requiring more than a month off and 4% requiring more than 6 months. Characteristics of injuries are presented in Table 2.

**Table 2:** Characteristics of the lacrosse injuries.

Location	All injuries^a^	Injuries^a^ in males	Injuries^a^ in females	Difference in proportions between males and females	Sex unspecified injuries
				[Pearson's *χ*^2^]	
	*n* = 1,138	*n* = 562	*n* = 563	*p*-value	*n* = 13
Head/neck	70	34	32	0.794	4
Eye	5	3	2	0.653	0
Face	16	9	7	0.612	0
Brain/concussion	34	19	15	0.483	0
Cervical spine	3	1	1	0.999	1
Other head injury	12	2	7	0.995	3
Trunk/back	130	73	54	0.072	3
Upper back	8	6	2	0.155	0
Chest	15	9	6	0.434	0
Abdomen	16	9	7	0.612	0
Lumbo-sacral spine	74	42	30	0.142	2
Groin	8	4	3	0.703	1
Buttock	9	3	6	0.317	0
Upper limb	169	**112**	57	**<0**.**001**	0
Shoulder	32	**22**	10	**0**.**031**	0
Arm	9	5	4	0.736	0
Elbow	8	**8**	0	**0**.**004**	0
Forearm	5	4	1	0.178	0
Wrist	29	**21**	8	**0**.**014**	0
Hand	30	18	12	0.265	0
Finger	26	15	11	0.425	0
Thumb	30	19	11	0.137	0
Lower limb	702	315	**383**	**<0**.**001**	8
Hip joint	28	12	16	0.447	0
Thigh	151	66	85	0.099	0
Knee	116	50	66	0.119	0
ACL	29	9	**20**	**0**.**039**	0
Lower leg	62	23	**39**	**0**.**037**	0
Achilles	3	2	1	0.562	0
Ankle	202	93	109	0.219	4
Ankle sprain	138	63	75	0.281	0
Toe	140	69	67	0.846	4
Time lost; *n*					
Yes	936	**481**	443	**0**.**003**	12
No	202	81	**120**	**0**.**003**	13
Severity					
Minimal (0 days lost)	173	65	**105**	**0**.**001**	3
Mild (1D-1W lost)	284	134	149	0.311	1
Moderate (1W-1M lost)	375	**199**	168	**0**.**046**	8
Severe (1M-6M lost)	259	141	117	0.086	1
Very severe (>6 months lost)	47	23	24	0.887	0
Type; *n*					
New	831	**429**	391	**0**.**009**	11
Recurrent	260	116	143	0.058	1
Exacerbated	47	17	29	0.072	1
Onset (part of the season); *n*					
Pre-season	655	314	333	0.267	8
In season	368	**203**	161	**0**.**007**	4
Post-season	97	39	57	0.056	1
Did not specify	18	6	12	0.155	0
Onset (match or training); *n*					
In match	187	**106**	81	**0**.**044**	0
In training	951	456	**482**	**0**.**044**	13
Mechanism; *n*					
Direct	425	**261**	161	**<0**.**001**	3
Indirect	84	48	36	0.171	0
Non-contact	629	253	**366**	**<0**.**001**	10
Fracture	121	**82**	**37**	**<0**.**001**	2
Overuse fracture	34	11	**21**	**<0**.**001**	2

Bold—highlights significant values; a *p*-value of < 0.05 was considered statistically significant.

^a^ The table depicts numbers per injury, not per athlete. Also, each athlete could report up to 3 injuries.

**Figure 1:**
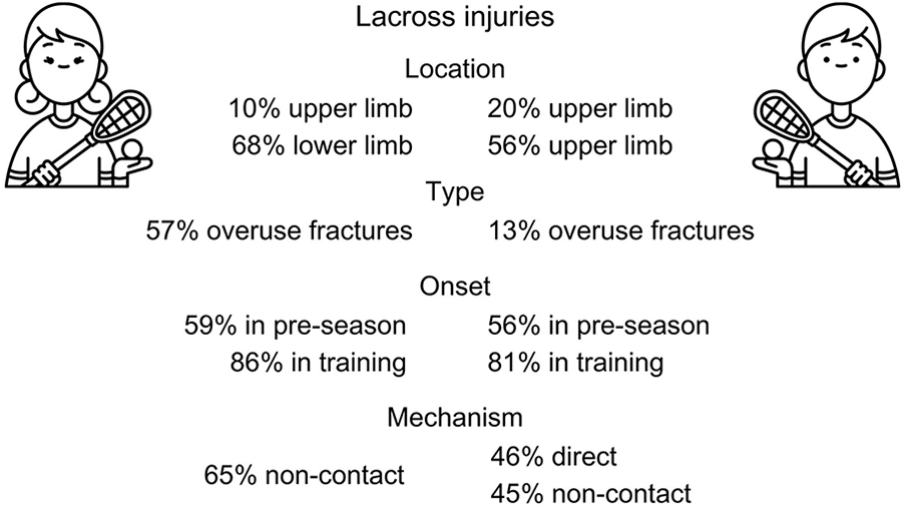
Differences in lacrosse injuries between females and males.

Among Japanese collegiate lacrosse athletes, males and females reported somewhat different patterns of injuries. A summary of the differences is presented in Figure 1. In females, 68% of injuries were to the lower limbs with 5% of these being ACL injuries, and only 10% being upper limb injuries. Fifty-seven percent of all fractures in this group were overuse fractures (vs. 13% in males). Most injuries in females happened in pre-season (59%), in training (86%), and due to a non-contact mechanism (65%). Males reported 20% of upper limb injuries and 56% of lower limb injuries. The majority of their injuries happened in training (81%) with 56% occurring in pre-season and 36% in season, due to direct (46%) or non-contact (45%) mechanisms.

Females and males differed in the proportions of some injury characteristics. Females had a significantly higher proportion of lower limb injuries, including ACL injuries and fractures but a lower proportion of injuries that required time off training or matches than males. A higher proportion of their injuries occurred in training and due to non-contact mechanisms. Compared to females, a higher proportion of males reported upper limb injuries, time lost due to an injury, new injuries, and injuries of moderate (1 week–1 month) severity, occurring in season, in match, and resulting from a direct mechanism. Half of the males and one-third of the females reported injury within the previous year but both groups had similar proportions of severe and very severe injuries (27%).

Injuries with severe impact included ACL injuries, fractures, and concussions. Out of 29 ACL injuries reported, 22 were sustained in pre-season, 4 in season, and 3 post-season, 21 ACL injuries occurred in training and 8 in match. The proportions of ACL injuries were similar between the athletes who had athletic trainers (*n* = 13) and not (*n* = 16). All ACL injuries were reported as severe or very severe, incurring mostly more than 6 months lost from training and competition. The severity distribution of ACL injuries was not different between females and males.

More than half (*n* = 70/121) of all fractures were severe (1–6 months lost) and one-fourth (*n* = 29/121) were moderate (1 week—1 month lost). Males had a significantly higher number of all fractures (82 fractures in males vs. 37 in females) but females had a higher proportion of overuse fractures (21 overuse fractures in females vs. 11 in males). More than half of overuse fractures (*n* = 19/34) were severe (1–6 months lost) and 7 were moderate (1 week to 1 month lost). Forty-one of the athletes who experienced fractures reported having an athletic trainer but 80 did not.

Half (*n* = 17/34) of concussions were of moderate severity (1 week to 1 month lost) but 7 were severe (1–6 months lost) and 9 were mild (1 day to 1 week lost). The frequency and severity proportions of concussions were not different between males and females.

### Injured vs. uninjured athletes

Differences in characteristics between injured and uninjured athletes are presented in Table 3. Significant differences in proportions between injured and uninjured athletes were found for sex, year at university, BMI, practice days per week, matches per season, and support from an athletic trainer. Additionally, group difference was found in age (injured 20.3 (±1.3) vs. uninjured 19.6 (±1.4); *t*-test, *p* < .003) and sports experience (injured 2.5 (±1.4) vs. uninjured 1.9 (±1.3); *t*-test, *p* < .004).

**Table 3:** Differences in characteristics between injured and uninjured athletes.

	Injured	Uninjured	Total	
	*n* = 700	*N* = 979	*N* = 1,679	
			*χ* ^2^	*P* value
Sex; *n* (%)				
Male	353 (50.4)	348 (49.6)	37.2	**<0** **.** **001**
Female	347 (35.5)	631 (64.5)		
Year at university; *n* (%)				
Year 1	94 (13.4)	456 (46.6)	205.8	**<0**.**001**
Year 2	222 (31.7)	205 (20.9)		
Year 3	136 (19.4)	126 (12.9)		
Year 4	248 (35.4)	192 (19.6)		
BMI; *n* (%)				
Underweight <18.5	58 (5.9)	35 (5.0)	8.1	**0**.**017**
Normal 18.5–24.9	865 (88.4)	600 (85.7)		
Overweight & Obese ≥25.0	56 (5.7)	65 (9.3)		
Practice days per week; *n* (%)				
1	4	2	83.0	**<0**.**001**
2	9	2		
3	142	36		
4	305	144		
5	480	468		
6	33	40		
7	6	8		
Matches/competitions per season; *n* (%)				
1–5 matches	682	385	41.0	**<0**.**001**
6–10 matches	193	204		
11–15 matches	52	54		
16–20 matches	26	29		
21–25 matches	14	13		
26–30 matches	2	7		
>30 matches	10	8		
Support available; *n* (%)				
Athletic trainer	431 (61.6)	462 (47.2)	33.9	**<0**.**001**
No athletic trainer	269 (38.4)	517 (52.8)		

Bold—highlights significant values; a *p*-value of < 0.05 was considered statistically significant.

### Factors related to an injury

Factors related to sustaining an injury are presented in Table 4. Male sex, higher years at the university, and support from an athletic trainer were identified as factors related with higher odds of sustaining an injury. Training five or more days per week was associated with lower odds of sustaining an injury.

**Table 4:** Factors related to sustaining an injury (logistic regression analysis).

	Odds ratio	95% CI	*P* value
Age	0.887	0.774–1.057	0.179
Sex			
Female	reference		
Male	**1** **.** **785**	1.394–2.285	**<0**.**001**
Year at university			
Year 1	reference		
Year 2	**5**.**484**	3.856–7.801	**<0**.**001**
Year 3	**6**.**046**	3.673–9.950	**<0**.**001**
Year 4	**8**.**000**	4.336–14.760	**<0**.**001**
Lacrosse experience	1.110	0.985–1.250	0.086
BMI			
Normal	reference		
Underweight	1.183	0.732–1.913	0.492
Overweight + Obese	0.977	0.644–1.484	0.914
Practice days per week			
1–3	reference		
4	0.865	0.550–1.361	0.531
5	**0**.**577**	0.374–0.889	**0**.**013**
6–7	**0**.**408**	0.212–0.786	**0**.**007**
Matches/competitions per season;			
1–5 matches	reference		
6–10 matches	0.668	0.814–1.378	0.668
>11 matches	0.878	0.614–1.255	0.475
Support			
No athletic trainer	reference		
Athletic trainer	**1**.**558**	1.240–1.957	**<0**.**001**

Bold—highlights significant values; a *p*-value of < 0.05 was considered statistically significant.

Additionally, mean time lost due to an injury (±SD) differed significantly between the athletes. Athletes (*n* = 716) who had the support of an athletic trainer missed 40.8 (±1.9) days, and athletes (*n* = 422) who did not, missed 33.6 (±2.3) days (*t*-test, *p*-value = 0.018).

## Discussion

The impact of injuries in Japanese collegiate lacrosse athletes is high for both females and males. Male sex, higher years at the university, and support from an athletic trainer were identified as factors related to higher odds of sustaining an injury. Training five, or more, days per week was associated with lower odds of sustaining an injury. This study complements the available reports on lacrosse injuries mostly in the American collegiate and high school populations and is a valuable contribution to the global lacrosse literature.

### Impact of injuries

The impact of the injuries in Japanese collegiate lacrosse athletes is high for both females and males. This is evident with severe and very severe injuries reported often for both groups with 1 in 4 injuries requiring more than a month off training and competition. Also, sports injuries with well-documented detrimental impacts are prevalent in the athletes in this study. Injuries to ACL reported by Japanese athletes appear to be more frequent (30 per year) than reported in the US collegiate lacrosse athletes (11–12 per year) (4, 14). These injuries also affected Japanese female athletes more (70% vs. 54%–56%) than previously reported in the comparable US population (4, 14). Overuse fractures reported in this study (3.7% of all injuries in females, 1.9% in males) were higher than reported for American high school athletes (2.8% for females, 1.5% for males) (15) but the fact that the proportion was higher in females seems to agree with previous reports for high school and collegiate US athletes (15, 16).

Head, brain, face, and eye injuries are of concern in lacrosse due to their potentially catastrophic consequences. In this study, concussions were 49% of all head injuries and were similarly frequent between males and females, whereas previous studies reported males sustain concussions more often than females (80% vs. 40%) (5). All concussions reported in this study required time off training which shows their detrimental impact. Eye injuries reported in this study were infrequent but considering the relatively small sample size and the self-reported nature of the study design, no conclusions can be made.

### Factors related to sustaining an injury

Male sex was a factor related to higher odds of sustaining injuries. Males reported more injuries and a higher proportion of their injuries was moderate in severity than females. This may be related to the more aggressive nature of the game for males than females, even though males use more protective equipment (2). The fact that male athletes' lacrosse injuries are at higher proportion new, of direct mechanism, and in season sustained during matches would support this claim. Also, these are in agreement with studies done on the American population both in terms of injury rates and characteristics (1, 3).

Higher year at university was related to higher odds of sustaining an injury. A low proportion of first-year student-athletes experienced injuries but starting with second-year athletes, the odds of sustaining injury increased markedly. This may be related to the fact that first-year students just start learning lacrosse and spend most of their time practicing technical and tactical drills rather than participating in matches, therefore having lower physiological demands imposed. This is supported by reported low experience in the sport. Also, some athletes may have had an experience in other sports to give them general athletic preparation and protect them from injuries in the first year when they are learning lacrosse as a new sport (17). This phenomenon was previously reported in the sample of first-year lacrosse female student-athletes, where those with previous sports experience in high school showed significantly different injury patterns than the ones for whom lacrosse was the first sport (17). Subsequently, from the second year onward, the sudden increase in training load due to the introduction of match play is likely occurring, exceeding the athletes' capacity, and making them susceptible to injury. The nature of the likely increased load should be investigated further. In the U.S., lacrosse players experience marked increase in the frequency of injuries from youth league to high school, and then again to collegiate level (6). As Japanese athletes often start participation in the lacrosse training in college, it is interesting to observe that their injury prevalence is still high even though their experience in the sport is not. The frequency of injuries is high in both American and Japanese collegiate athletes, but the reasons must go beyond the age. In American collegiate female lacrosse players, injuries were related to the high intensity of play at this age group and their anthropometric characteristics (6). Also, higher frequency of in game vs. in training injuries seem to be true for both U.S. and Japanese players. The mechanism of lacrosse injuries was described mainly as incidental contact (e.g., stick-to-player or player-to-ball contact) in American female lacrosse players (6) but we found non-contact mechanism dominating in females of this study. Therefore, although the frequency of injuries is high in both American and Japanese populations, the mechanism and nature of these injuries differ leading to different injuries. This finding may suggest the need for development of different injury prevention strategies in these two groups. The need for culturally adapted approaches to injury prevention starts to emerge from the literature at the moment (18), and this study seems to confirm this need.

The support of an athletic trainer was a related factor with increased both the number and severity of injuries in lacrosse athletes. This finding conflicts with studies reporting the viability of athletic trainer use for injury prevention, management, and cost-effectiveness in other sporting populations or the crucial role athletic trainers play in prevention of sudden death in sport (19–21). The increase in the number and severity of injury found in this study could be attributed to two possibilities: (1) a true increase in the number and severity of injuries or (2) increased reporting. If the found association is truly present, the factors explaining it could relate to the athletic trainers using a variety of techniques (e.g., taping) to enable an athlete to participate in the match or training with an existing injury, possibly causing the injury to deteriorate for the benefit of participation. The care of athletic trainers may also allowed athletes to play more with suboptimal health and therefore allow for the training loads to exceed athlete's capacity to recover. Moreover, athletic trainers could have reviewed and postponed the return to play of their athletes after an injury, leading to higher severity scores in this study. If, on the other hand, the found outcome is only present due to improved reporting, it could be related to athletic trainers sending the athletes for medical consultation, leading to early diagnosis and treatment as reported previously (22). Another aspect could be related to the signs and symptoms' education provided by athletic trainers that could lead to better injury reporting by the athletes. Previous studies have shown mixed findings on this matter with some showing support of athletic trainers leading to higher frequency of reporting of sport-related concussions (23, 24). We cannot exclude an influence of face-to-face interaction the athlete had with an athletic trainer (or other health professional) that may have led to better recall and reporting. Lastly, having the support of an athletic trainer could have normalized talking about injuries, which in Japan is traditionally avoided due to the strong culture of dedication to the sport no matter what, building toughness and resilience in athletes. These speculated behaviors need further investigation to explain our findings and subsequently optimize athletic trainer's role and influence in the Japanese collegiate lacrosse population.

Contrary to previously discussed factors, the number of practice days of at least 5 per week was associated with lower odds of sustaining an injury. The training load applied over the 5 days may have allowed for appropriate physical preparation and optimal acquisition of technical skills, that would protect the athletes from sustaining injuries. Whereas the athletes who trained less than 5 days could suffer from undertraining and under preparation, leading to higher susceptibility to getting injured especially later in their carrier (higher year at the university). This finding agrees with the contemporary science of training load saying that a high chronic training load may have a protective effect against injuries (25). As mentioned before, investigation into training load in lacrosse population is warranted.

### Limitations

Following limitations should be considered. Firstly, this study had a survey design with self-selected participants. The respondents who voluntarily choose to participate in the survey may not represent the entire population accurately. This self-selection bias could affect the external validity of the study. Moreover, non-response bias could arise if certain individuals within the sample chose not to participate, leading to a lack of representation and potential bias in the study's outcomes.

Second limitation is the retrospective nature of the study, which may be susceptible to recall bias. Participants were asked to recall past events, which could be influenced by memory limitations or personal biases. Moreover, the injury diagnosis was self-reported by athletes, and could have been less accurate than in person assessment. To minimize the impact, medical doctor verified all entries to assure that the data entered by athletes were probable. The observational nature of the study design limits the ability to establish causality between variables.

Finally, it is important to note that the findings of this study might not be applicable to other contexts or populations. Factors such as cultural differences, geographic location, or time period might affect the generalizability of the results. Caution should be exercised when extrapolating these findings to different settings or populations without considering these contextual factors.

### Recommendations

Even though males' lacrosse rules were modified in the hope to reduce the injuries, males in this study were 1.8 times more likely to sustain an injury than females. Further efforts aimed specifically at this group should be warranted.

The results of this study suggest that time spent on practice (physical, technical) at least 5 days per week reduces the risk of sustaining an injury. Further research is needed to establish what exact explanation for this finding is (e.g., volume, frequency). Similarly, attention should be put into long term lacrosse athlete development in collegiate population, as to prepare the athletes for the demands of the sport appropriately.

Another recommendation would be to investigate in detail how to improve an athletic trainer's support practices to ensure that it is benefiting athletes' health in both the short- and long-term.

## Conclusions

Male sex, higher year at the university, and support from an athletic trainer were identified as factors related to higher odds of sustaining an injury, while practicing at least 5 days per week was a protective factor. These findings provide a new inside into lacrosse injury knowledge as some of them are novel or contrary to the previously reported mainly in the US population.

## Data Availability

The raw data supporting the conclusions of this article will be made available by the authors, without undue reservation.
